# Accuracy of a Machine-Learning Algorithm for Detecting and Classifying Choroidal Neovascularization on Spectral-Domain Optical Coherence Tomography

**DOI:** 10.3390/jpm11060524

**Published:** 2021-06-08

**Authors:** Andreas Maunz, Fethallah Benmansour, Yvonna Li, Thomas Albrecht, Yan-Ping Zhang, Filippo Arcadu, Yalin Zheng, Savita Madhusudhan, Jayashree Sahni

**Affiliations:** 1 Pharma Research and Early Development, Roche Innovation Center, F. Hoffmann-La Roche Ltd., 4070 Basel, Switzerland; fethallah.benmansour@roche.com (F.B.); yvonna.li@roche.com (Y.L.); tom.albrecht@roche.com (T.A.); yan-ping.zhang_schaerer@roche.com (Y.-P.Z.); filippo.arcadu@roche.com (F.A.); jayashreesahni@yahoo.co.uk (J.S.); 2 Department of Eye and Vision Science, University of Liverpool, Liverpool L7 8XP, UK; Yalin.Zheng@liverpool.ac.uk (Y.Z.); Savita.Madhusudhan@liverpoolft.nhs.uk (S.M.); 3 Liverpool Ophthalmic Reading Centre (NetwORC, UK), St. Paul’s Eye Unit, Royal Liverpool University Hospital, Liverpool L7 8XP, UK

**Keywords:** age-related macular degeneration, choroidal neovascularization, classification, machine learning, optical coherence tomography

## Abstract

Background: To evaluate the performance of a machine-learning (ML) algorithm to detect and classify choroidal neovascularization (CNV), secondary to age-related macular degeneration (AMD) on spectral-domain optical coherence tomography (SD-OCT) images. Methods: Baseline fluorescein angiography (FA) and SD-OCT images from 1037 treatment-naive study eyes and 531 fellow eyes, without advanced AMD from the phase 3 HARBOR trial (NCT00891735), were used to develop, train, and cross-validate an ML pipeline combining deep-learning–based segmentation of SD-OCT B-scans and CNV classification, based on features derived from the segmentations, in a five-fold setting. FA classification of the CNV phenotypes from HARBOR was used for generating the ground truth for model development. SD-OCT scans from the phase 2 AVENUE trial (NCT02484690) were used to externally validate the ML model. Results: The ML algorithm discriminated CNV absence from CNV presence, with a very high accuracy (area under the receiver operating characteristic [AUROC] = 0.99), and classified occult versus predominantly classic CNV types, per FA assessment, with a high accuracy (AUROC = 0.91) on HARBOR SD-OCT images. Minimally classic CNV was discriminated with significantly lower performance. Occult and predominantly classic CNV types could be discriminated with AUROC = 0.88 on baseline SD-OCT images of 165 study eyes, with CNV from AVENUE. Conclusions: Our ML model was able to detect CNV presence and CNV subtypes on SD-OCT images with high accuracy in patients with neovascular AMD.

## 1. Introduction

Early detection of active choroidal neovascularization (CNV) is crucial for the timely treatment of neovascular age-related macular degeneration (nAMD), in order to achieve a good outcome [1]. Clinicians are increasingly switching from fluorescein angiography (FA) to optical coherence tomography (OCT) for the diagnosis and management of nAMD, due to the advantages associated with OCT, including being noninvasive, enabling quick acquisition of retinal images with minimum technician training, and providing both qualitative and quantitative information [2,3,4]. However, an advantage of FA is that it provides information on flow dynamics within the lesion [5], and most importantly, confirms disease activity, characterized by dye leakage. Phenotyping CNVs at baseline on FA, and sometimes additionally on indocyanine green angiography, has long been the standard of care and helps establish the management plan. For example, patients with polypoidal choroidal vasculopathy (PCV) may benefit from combination therapy [6,7,8]. In clinical trials, this information would help identify subgroups of patients with particularly beneficial outcomes, with novel therapies [9]. Optimal patient stratification may become increasingly important, as multiple combination therapies are poised to enter the market or are in clinical trials, especially in patients who show partial response or non-response to current first-line treatment with antiVEGF agents [10,11].

In OCT, CNV is graded based on its relationship to the retinal pigment epithelial layer [12,13], whereas in FA, en-face flow patterns within the lesion are used to phenotype the CNV [13]. By comparing the two modalities and using an automated approach to evaluate the large quantity of data from three-dimensional SD-OCT volume scans, key features mirroring the flow dynamics and substituting en-face information available in FA could be extracted. Once identified, the impact of novel and existing therapies on these key features could increase our understanding of the disease phenotype, pathophysiology, and specific response to therapy.

Machine learning has the potential to unravel high-dimensional patterns from image data (complex interactions related to a given phenotype), as opposed to features that are correlated only individually to the outcome, providing enhanced capabilities for knowledge extraction. It also provides options for automated screening and diagnosis, enhancing the speed and reproducibility of these processes.

In this study, using the phenotypic CNV definitions derived from FA as the reference standard, we developed a machine learning (ML) model capable of identifying these CNV subtypes, using OCT alone. We present the data on performance of this model for the detection and classification of CNV (as per FA) using the SD-OCT images. In addition, using a sub-symbolic approach, we identified key features on OCT that relate to particular CNV subtypes on FA. To the best of our knowledge, no previous study has leveraged this combination of ML approaches or reported findings similar to those presented in the current study.

## 2. Materials and Methods

### 2.1. Participants

This study was a retrospective analysis of prospectively collected baseline FA and SD-OCT images of the study eyes and fellow eyes of patients with nAMD, in the phase 3 HARBOR (NCT00891735) and phase 2 AVENUE (NCT02484690) trials. 

The above trials adhered to the tenets of the Declaration of Helsinki, were Health Insurance Portability and Accountability Act compliant, and the protocols were approved by the relevant institutional review boards and ethics committees. Patients provided written informed consent for secondary use of data at enrolment, including future medical research, and additional analyses. In HARBOR, SD-OCT was performed using the Cirrus HD-OCT III instrument (Carl Zeiss Meditec, Dublin, CA, USA) producing 512 × 128 × 1024 voxels with a size of 11.7 × 47.2 × 2.0 µm^3^, covering a volume of 6 × 6 × 2 mm^3^. In AVENUE, SD-OCT was performed using the Heidelberg Spectralis instrument (Heidelberg Engineering, Heidelberg, Germany). Study design and main outcomes of HARBOR [14,15] and AVENUE [10] have been published previously. In brief, both studies recruited patients with treatment-naive subfoveal CNV secondary to AMD, as diagnosed by a reading center (Digital Angiography Reading Center, Great Neck, NY [DARC]). In AVENUE, patients with juxtafoveal CNV on FA, with a subfoveal component on SD-OCT were also included. Eligibility for both studies was confirmed by the same central reading center (DARC), and the published standard definitions of CNV types have been used in both studies [13].

### 2.2. Classification of CNV

Both studies allowed recruitment of all CNV types. CNVs were classified at baseline on FA as predominantly classic, minimally classic, or occult, based on the proportion of the occult component within the CNV lesion, as previously described by the Macular Photocoagulation Study (MPS) Group [13].

### 2.3. Selection of Fellow Eyes

Treatment-naive fellow eyes of patients in HARBOR without advanced AMD, were also included to train the model. Fellow eyes were filtered to exclude those with prior treatment or any type of late-stage AMD. 

### 2.4. OCT Image Processing and Analysis

#### 2.4.1. Retinal Layer Segmentations

**Figure 1:**
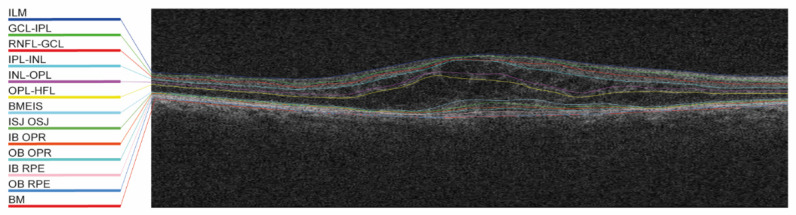
Example of the automated layer segmentation of 13 retinal layers. BM, Bruch’s membrane; BMEIS, boundary of myoid and ellipsoid inner segments; GCL-IPL, ganglion cell layer-inner plexiform layer; IB OPR, inner boundary outer photoreceptor; IB RPE, inner boundary retinal pigment epithelium; ILM, internal limiting membrane; INL-OPL, inner nuclear layer-outer plexiform layer; IPL-INL, inner plexiform layer-inner nuclear layer; ISJ OSJ, inner segment/outer segment junction; OB OPR, outer boundary outer photoreceptor; OB RPE, outer boundary retinal pigment epithelium; OPL-HFL, outer plexiform layer-Henle’s fiber layer; and RNFL-GCL, retinal nerve fiber layer-ganglion cell layer.

Twelve retinal layers (Figure 1; among them inner limiting membrane and retinal pigment epithelium [RPE]) were automatically segmented in all selected SD-OCT volumes using the Iowa reference algorithm [16]. Bruch’s membrane was added as the thirteenth layer (convex hull of the RPE) and was computed using scikit-image [17]; thus, it was based on an approximation and not on a real segmentation.

#### 2.4.2. Fluid Annotations 

Fluid volumes were annotated by experts from the Liverpool Ophthalmology Reading Center on the B-scan level and subjected to internal quality assurance processes (a subselection of B-scan annotations of each grader was adjudicated and reviewed by a senior clinician). Specifically, a sparse selection of 19 B-scans per volume scan across a total of 50 volume scans (950 B-scans in total), obtained from Cirrus (Carl Zeiss Meditec) OCT machines (see Supplementary Table S1 for definitions used for the annotations), were annotated by drawing contours of the intraretinal fluid (IRF; cystoid spaces), subretinal fluid (SRF), and pigment epithelial detachment (PED), as well as subretinal hyperreflective material (SHRM; a morphological feature seen on OCT as hyperreflective material located external to the neurosensory retina but internal to the RPE [18,19]).

Contours were drawn on the B-scans, stored in the raster format, and then converted to label maps of the original image dimension. The annotations were done using a Matlab software tool developed for the Liverpool reading center. 

#### 2.4.3. Fluid Segmentations

The U-Net, a convolutional neural network for biomedical image segmentation [20], was trained to recognize fluids, using the annotated volumes as a training material (pixel-level semantic segmentation). All SD-OCT volumes from both HARBOR and AVENUE were segmented with the trained U-Net model, and there was no adaptation of the model to accommodate the specific characteristics of the AVENUE volumes (Heidelberg Spectralis).

#### 2.4.4. Feature Generation 

**Figure 2:**
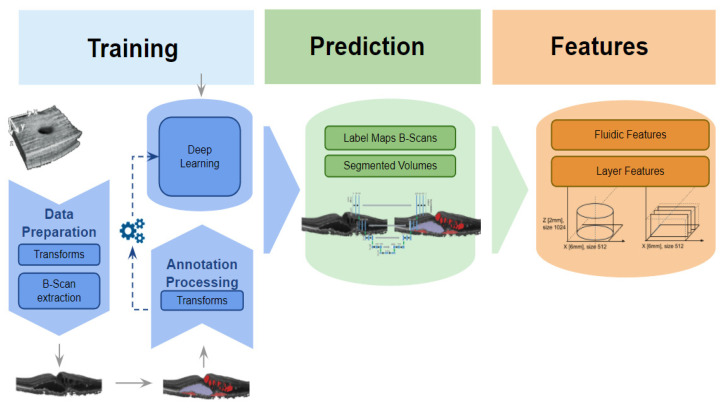
Segmentation pipeline. Sketch of the segmentation pipeline, involving training, prediction, and feature calculation for both fluidic and layer features. See Supplementary Table S2 for a detailed feature list.

In total, 105 volume and volume-wide thickness descriptors (see Supplementary Table S2 for a detailed feature list), based on the macular subfield definitions provided by the Early Treatment Diabetic Retinopathy Study [21] grid, were automatically extracted from the automated segmentations. See Figure 2 for a sketch of the segmentation and feature extraction pipeline. Specifically, IRF, SRF, and PED, as well as SHRM, were segmented using the segmentation method described above, then reassembled into volumes, where each voxel is mapped to either one of the four segmented targets, or not mapped to any. Finally, descriptors were first individually derived for all B-scans of a volume, and then combined to form C-scan volume measurements.

#### 2.4.5. Machine Learning

The SD-OCT features were profiled for their utility to predict various binary outcomes (see the Results section for outcome definitions) derived from FA. Cross-validation was used to assess the predictive performance. When no hyperparameter tuning or feature selection was used, no holdout set was put aside and every data point was predicted exactly once. The corresponding performance was reported. This approach was used for all outcomes that reached very high area under the receiver operating characteristic (AUROC) values, namely the CNV presence/absence outcome. Otherwise, a holdout set with 15% of the data was put aside first, and supervised feature elimination and hyperparameter tuning were performed in two stages, using non-nested cross-validation on 85% (Supplementary Figure S1) of the imaging data. A model was established using the best feature sets and the hyperparameter values found, and the predictive performance on the holdout set was subsequently reported.

When classifying predominantly classic versus occult CNV, the tuning process described above was applied to 100% of HARBOR data, and the established model was used to predict predominantly classic and occult CNV on SD-OCT of AVENUE study eyes (Supplementary Figure S2). The predictions were then compared with the FA labeling in AVENUE for accuracy. See the Supplementary Methods for details on the ML methodology.

### 2.5. Statistical Analysis 

#### 2.5.1. Classification

AUROC was used to measure performance of the models to discriminate two classes from each other. AUROC is a measure of discriminative performance of a binary classifier predicting a numeric score (probability) for class membership. AUROC was obtained by sliding a threshold over the predicted probability to assess the tradeoff between sensitivity and specificity learned by the model. Each point thus obtained was associated with a certain sensitivity and specificity. Reported pairs of sensitivity and specificity corresponded to Youden’s cutoff point. The receiver operating characteristic (ROC) curve was plotted with sensitivity and specificity on the y- and x-axes, respectively. The perfect classifier will have sensitivity and specificity = 1, and the ROC curve will pass through the top left corner of the chart, with an AUROC = 1. Confidence intervals for ROC values were obtained by bootstrapping.

#### 2.5.2. Correlations

To validate the automatically generated features, non-parametric Spearman correlation coefficients were used, measuring the correlations between the reading center reads and an associated subset of the automated image analysis features.

#### 2.5.3. SHapley Additive exPlanations (SHAP) Analysis

SHAP analysis [22] is a means to analyze individual predictions made by an ML model. For each feature and predicted data point, SHAP analysis explains the difference between the average model prediction of a given dataset and the individual prediction of this point. This approach explains individual predictions and contributions of each feature, as well as analyzes the overall significance (impact and bias) of the features in a trained ML model, with respect to a given population.

## 3. Results

### 3.1. Patient Characteristics

Out of a total of 1098 patients randomized in HARBOR, baseline FA and SD-OCT images were available for 1037 study eyes. In the HARBOR baseline data, CNVs were classified (per FA) as predominantly classic in 163 eyes, minimally classic in 492 eyes, and occult in 382 eyes. Out of a total of 272 patients randomized in AVENUE, baseline FA and SD-OCT images were available for 268 study eyes. CNVs in AVENUE at baseline were classified (per FA) as predominantly classic in 39 eyes, minimally classic in 103 eyes, and occult in 126 eyes. Additionally, 531 healthy fellow eyes from HARBOR without advanced AMD (neither CNV nor GA) were used as negative controls.

### 3.2. Feature Generation

Segmentation performance for SRF, IRF, PED, and SHRM was assessed against annotations on the HARBOR SD-OCT images and was measured via DICE scores (Sørensen–Dice similarity coefficients; Table 1). Performance for SHRM and PED was better than that for SRF and IRF. Feature evaluation was also performed on various thickness and volumetric reads from AVENUE. Automatically extracted features with the closest definitions to the reading-center-defined feature readouts were selected for comparison to the manual reads, in order to demonstrate that there was a high correlation between manual readouts and automated readouts (Table 2). 

Type indicates volumetric pathology type; *N* (Train/Valid) indicates number of samples in the training and validation datasets, respectively; validation DICE indicates the DICE score achieved during the validation of the model.

IRF, intraretinal fluid; PED, pigment epithelial detachment; SD, standard deviation; SHRM, subretinal hyperreflective material; and SRF, subretinal fluid.

Various thickness and volumetric measurements compared to the reading center readouts.

BM, Bruch’s membrane; IB, inner boundary; ILM, inner limiting membrane; Spearman r, Spearman correlation coefficient; PED, pigment epithelial detachment; RPE, retinal pigment epithelium; and SRF, subretinal fluid.

Balance indicates the number of positive/negative cases; cutoff indicates the critical value of the predicted score corresponding to the AUROC value.

AUROC, area under the receiver operating characteristic; CNV, choroidal neovascularization; FA, fluorescein angiography; FN, number of false negatives; FP, number of false positives; and SD-OCT, spectral-domain optical coherence tomography.

### 3.3. HARBOR Analysis

**Figure 3:**
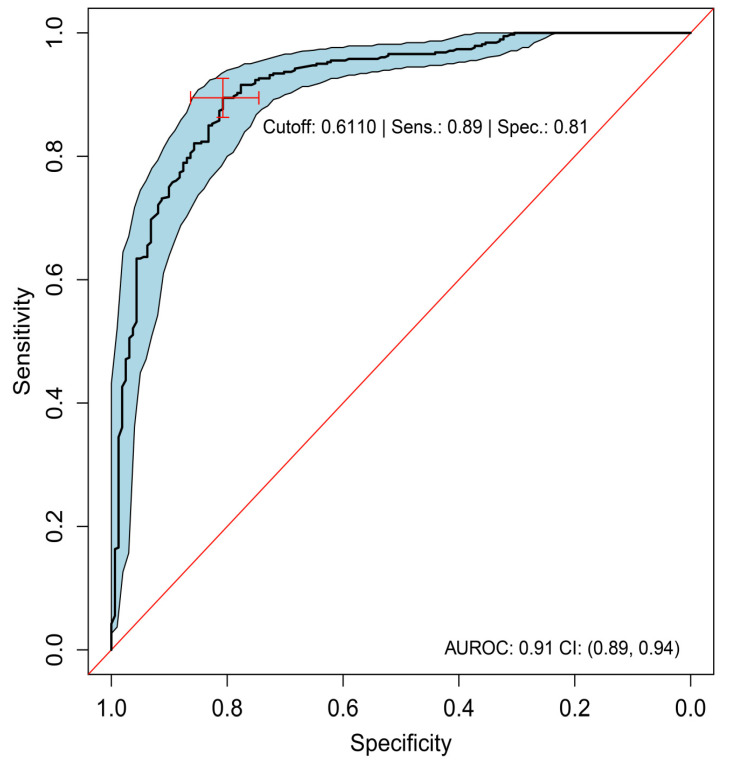
ROC analysis of predominantly classic versus occult (best-tuned performance). Sensitivity versus specificity for all possible ROC cutoff points with respect to the predicted occult scores in HARBOR, including 95% CIs (bootstrapped). The location of the red crosshair indicates the operating point of the model. AUROC, area under the receiver operating characteristic; FA, fluorescein angiography; NEG, negative; POS, positive; ROC, receiver operating characteristic; Sens, sensitivity; and Spec, specificity.

As described in the section ‘Machine Learning’, we assessed the ability of ML to discern eyes with any CNV type from eyes without CNV on SD-OCT, by pooling data across the three types (predominantly classic, minimally classic, and occult) and contrasting them with feature data from the 531 healthy fellow eyes. Presence of any CNV could be almost perfectly discriminated from absence of CNV (AUROC, 0.99; 95% CI, 0.99–1.00; Table 3). Occult and predominantly classic CNV could be discriminated with high accuracy from each other with AUROC = 0.91 (95% CI, 0.89–0.94; Figure 3, Table 4). Specificity for discrimination of occult from a predominantly classic CNV was 81%, with a sensitivity of 89%, when defining occult as the positive class and predominantly classic as the negative class. There were 32 false positives and 41 false negatives out of 163 actually negative and 382 actually positive observations, respectively (Table 5). Minimally classic was discriminated from occult and predominantly classic with AUROC = 0.70 (95% CI, 0.60–0.79) and AUROC = 0.73 (95% CI, 0.61–0.85), respectively. Occult was discriminated from minimally classic and predominantly classic (when the two were pooled together) with AUROC = 0.81 (95% CI, 0.73–0.88).

ROC indicates area under the curve in percentage; and resample indicates the specific fold from the five-fold cross-validation.

Predicted indicates class predicted by the model; observed indicates class as graded on FA; and *n* indicates the number of samples.

### 3.4. Recursive Feature Elimination

**Figure 4:**
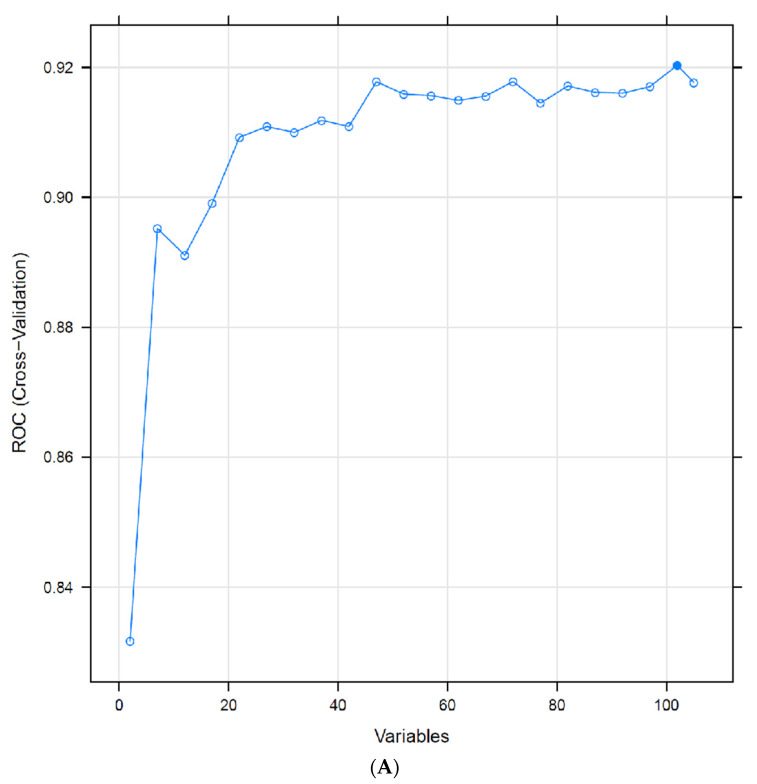

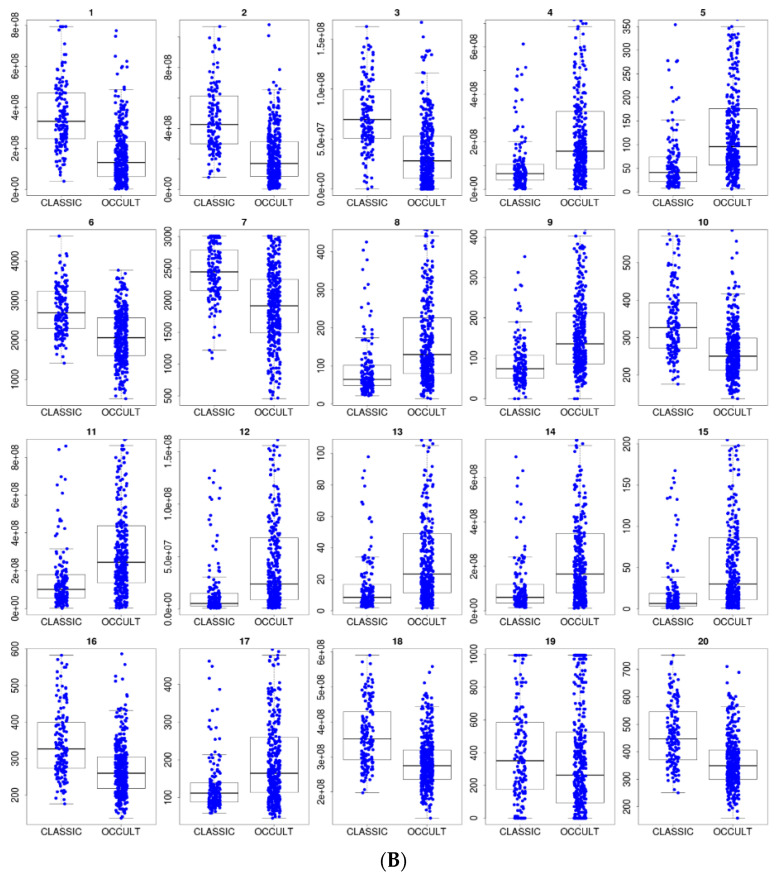
(**A**) Recursive feature elimination cross-validation. Optimal performance for predominantly classic versus occult was reached with 101 features out of 106, and only 21 features were necessary to sustain the average model performance of 91% AUROC. (**B**) Distribution of the top 20 feature values in the training data (predominantly classic vs. occult classes).

**Figure 5:**
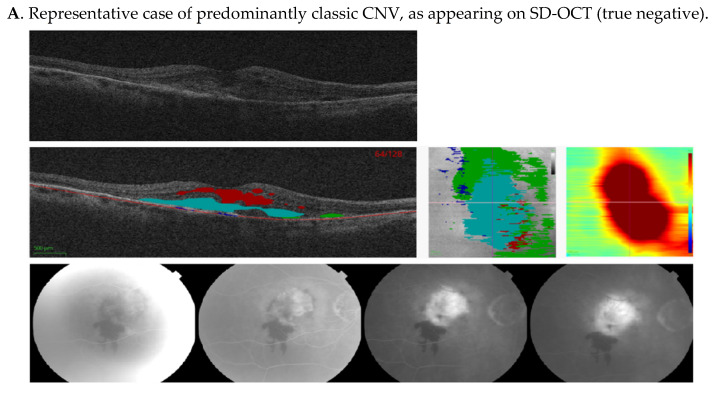

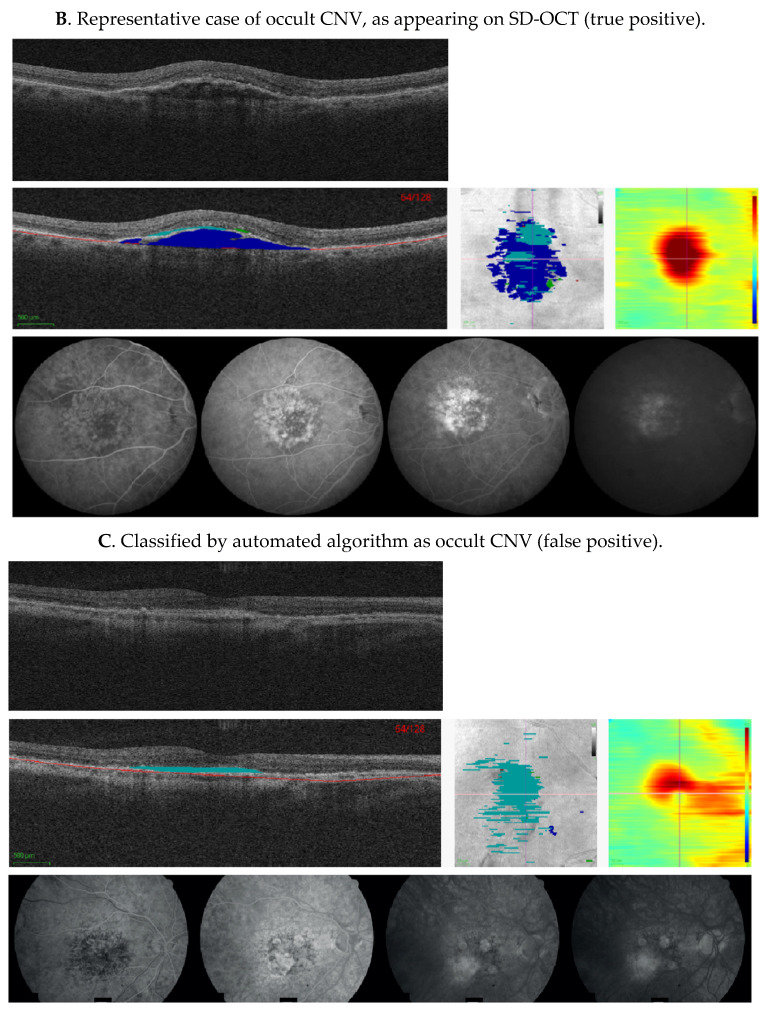

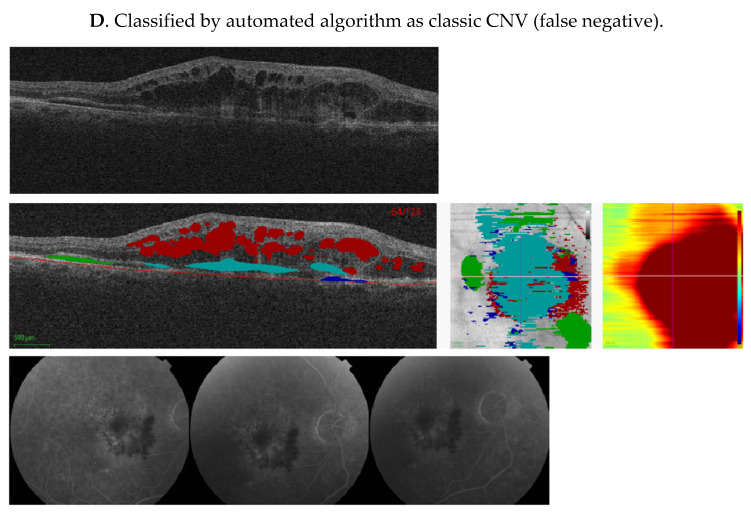
Representative cases showing comparison of machine algorithm with angiography. (**A–D**) Central SD-OCT B-scans (top), with segmented pixel masks of volumetric measures and Bruch’s membrane (middle left), en-face projections (middle center), and thickness maps (middle right), as well as corresponding FAs (bottom). Colors on the SD-OCT images indicate volumetric measures as follows—intraretinal fluid (red), subretinal fluid (green), PED (blue), and SHRM (cyan). Bruch’s membrane is shown as a red line. In (**A**), FA shows an area of hypofluorescence due to hemorrhage, and a well-demarcated area of hyperfluorescence due to a predominantly classic CNV that leaks in later frames. This was also identified as classic CNV by our ML algorithm, due to increased SHRM height and volume. In (**B**), FA demonstrates an ill-defined area of stippled hyperfluorescence, due to an occult CNV that leaks diffusely in mid and late frames, and was also identified as occult CNV by the ML algorithm, due to the presence of the PED. In (**C**), FA shows an area of well-defined hyperfluorescence in mid frames that stains and leaks in late frames due to fibrosis. The image was classified as classic CNV by the reading center, but was identified as occult CNV by the ML algorithm due to low SHRM height and volume. In (**D**), FA shows an area of hypofluorescence due to hemorrhage and a poorly demarcated area of hyperfluorescence due to the CNV. This lesion was defined as minimally classic by the reading center, but was identified as classic CNV by the ML algorithm due to the SHRM created by the hemorrhage. CNV, choroidal neovascularization; FA, fluorescein angiogram; ML, machine learning; PED, pigment epithelium detachment; SD-OCT, spectral-domain optical coherence tomography; and SHRM, subretinal hyperreflective material.

**Figure 6:**
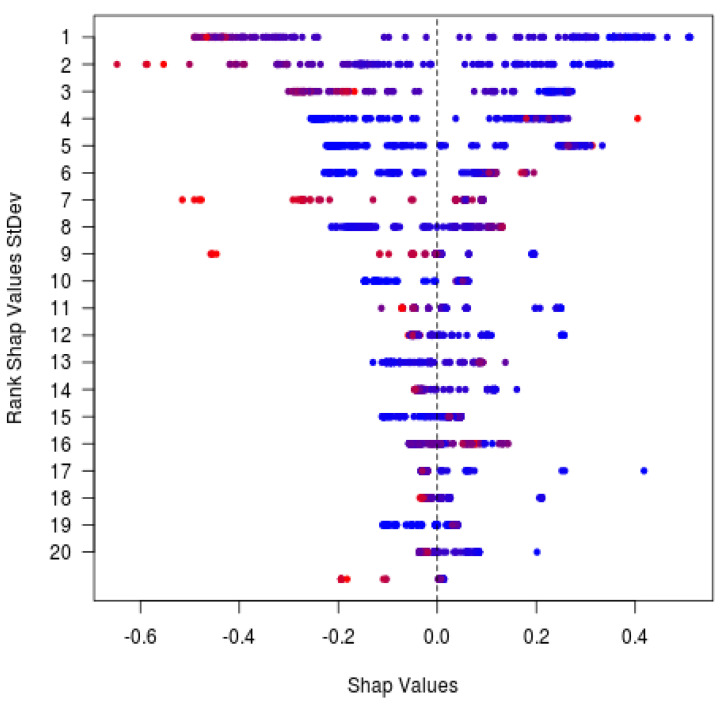
SHAP analysis external validation. SHAP analysis for the CNV type predictions in AVENUE. Every prediction contributes exactly one dot to each row. Blue and red colors indicate lower and higher feature values, respectively. SHAP values (x-axis) add up to the predicted probability for occult (only 20 features with highest SHAP variance shown here). BM, Bruch’s membrane; CNV, choroidal neovascularization; HFL, Henle’s fiber layer; IB, inner boundary; ILM, inner limiting membrane; IRF, intraretinal fluid; max, maximum; OB, outer boundary; OPL, outer plexiform layer; PED, pigment epithelial detachment; RPE, retinal pigment epithelium; SHAP, SHapley Additive exPlanations; and SHRM, subretinal hyperreflective material.

For discrimination of occult from predominantly classic CNV, when starting with the full 105-feature set and recursively eliminating the least important features, by repeatedly cross-validating with the reduced set, only 21 features were necessary to sustain an average AUROC = 0.91 (Figure 4A). The 20 most informative features for discrimination between occult and predominantly classic on a population level include SHRM and PED volumes (Figure 4B, Table 6). See Figure 5A,B for representative appearance of occult and predominantly classic cases on SD-OCT. SHAP analysis (Figure 6, Table 7) indicates that higher values of SHRM volumes and lower values of PED volumes are most characteristic of predominantly classic CNV, as opposed to occult CNV. Note the largely overlapping findings with feature elimination in Table 6.

### 3.5. External Validation Using AVENUE Data 

**Figure 7:**
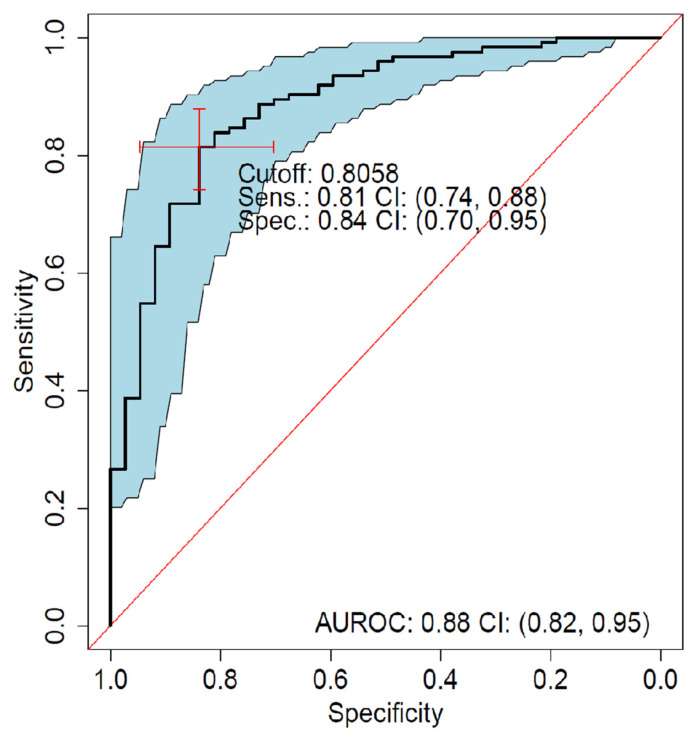
ROC analysis of predominantly classic versus occult external validation. Sensitivity versus specificity for all possible cutoff points with respect to predicted occult scores in AVENUE, including 95% CIs (bootstrapped). The location of the red crosshair indicates the operating point of the model. AUROC, area under the receiver operating characteristic; FA, fluorescein angiography; NEG, negative; POS, positive; ROC, receiver operating characteristic; Sens, sensitivity; and Spec, specificity.

The performance of the model for differentiating predominantly classic from occult CNV on SD-OCT images of AVENUE was AUROC = 0.88 (95% CI, 0.79–0.94; Figure 7). Specificity was 84%, with sensitivity of 81%, when defining occult as the positive class and predominantly classic as the negative class. There were seven false positives and 24 false negatives out of 39 actually negative and 126 actually positive observations, respectively (Table 8). The most important features (according to the model internal measures) for detection of CNV were SHRM and PED (Figure 6 and Table 7).

## 4. Discussion

Until recently, FA was the reference standard to establish the diagnosis of nAMD and sometimes is also used to monitor patient response to treatment, by assessing reduction in leakage or CNV area [5,23]. However, due to the ease of image acquisition and interpretation, OCT has become the modality of choice for monitoring disease course in clinics [2,3,4]. As the two imaging modalities use different features and provide different data, here, we presented a bridging study that used data generated on FA to identify and subclassify CNV on SD-OCT, using ML with high accuracy. The availability of two independent sets of large and well-characterized data from the HARBOR and AVENUE clinical trials with well-defined inclusion and exclusion criteria, standardized protocols for acquisition of images, and grading of CNV, allowed the development and robust external validation of our model. 

Our ML SD-OCT algorithm was trained using FA-based classification of CNV. This algorithm, developed using Zeiss Cirrus OCT images, was able to discriminate CNV absence versus CNV presence with very high accuracy (99%) and subclassify occult from predominantly classic CNV subtypes, with an accuracy of 91% AUROC. Furthermore, the performance accuracy of the ML algorithm using an external dataset was 88%, despite it being a different SD-OCT machine (Heidelberg Spectralis). Accuracy of FA-versus OCT-based approaches for detection of fluid has been explored by several researchers [24,25,26], but few have attempted to bridge the two technologies for the identification and classification of CNV [27,28,29,30,31]. Using FA as the reference standard for identification and classification of CNV, Wilde et al. [31] retrospectively evaluated 278 eyes diagnosed with CNV on SD-OCT, with their corresponding FA. They reported that while sensitivity of SD-OCT in detection of CNV was high (100%), it had a low specificity, with a 17% false-positive rate. Their findings were similar to other studies [26,28,30] that evaluated leakage on FA as a surrogate marker for CNV activity and found the sensitivity of SD-OCT to be high, but lacking specificity in comparison. Limited details of criteria for CNV identification by SD-OCT are provided in this publication, and it appeared that decision-making was mainly based on subjective criteria; features such as SHRM and PED were not included in the analysis of SD-OCT.

Our algorithm well-differentiated between occult and predominantly classic CNV types, whereas the ability to differentiate minimally classic CNV from occult or predominantly classic was lower. Our model identified the most informative features for discrimination between occult and predominantly classic, such as SHRM and PED volumes; higher SHRM volumes and lower PED volumes were most characteristic of predominantly classic CNV, as opposed to occult CNV. In contrast, absence of a well-defined SHRM in a case classified as classic CNV on FA was diagnosed as occult CNV by our model (Figure 5C), and the appearance of SHRM due to hemorrhage, resulted in our model identifying it as predominantly classic CNV, while the lesion was classified by the reading center as occult on FA (Figure 5D). Minimally classic CNV by definition has lesion components of both classic and occult CNV [13]. As the algorithm learns to find salient characteristics for either class during training, a class that combines characteristics of two other classes (instead of having its own characteristic features), posed an intrinsically harder problem to discriminate [13].

There are only a few publications that have attempted to correlate FA-defined phenotypes of CNV with features of CNV on OCT [27,29]. In a recent study by Gualino et al. [29], five retina specialists compared SD-OCT combined with color fundus photography or FA in 148 patients with treatment-naive nAMD. They classified CNV as type 1, 2, or ‘other CNV’, based on study-defined prespecified criteria, including features such as PED and SHRM in their decision-making. Manual readouts performed using subjective criteria developed specifically for these manuscripts/studies limit the wider application of their findings. However, it is interesting and reassuring to note that our algorithm developed on FA takes the same features into consideration as human graders in this study, to classify lesion types into type 1 and 2 on SD-OCT [12,29]. The strength of our approach is that, it is completely automated on SD-OCT and that we used the MPS standardized FA classification as the base for classification of CNV [32]. Applied to clinical practice, this automated diagnostic SD-OCT-only process may help to expand the population of patients that can benefit from CNV assessment, for example, in remote environments, without easy access to a retinal center with multiple imaging modalities.

We also presented segmentation performance for the various OCT features. For IRF and PED, the performance was poor at 0.46 (±0.12) and 0.63 (±0.07), respectively, whereas for SRF, it was better (0.67 [±0.05]); see Table 1. Interestingly, for SHRM, the model had good performance (0.71 [±0.06]). In the RETOUCH grand challenge, segmentation performance ranged between 0.57–0.85 for IRF, 0.54–0.72 for SRF, and 0.66–0.82 for PED in terms of DICE score [33]. SHRM segmentation was not part of the RETOUCH challenge. In our study, SHRM performance was assessed using a subset of our annotations. The reduced performance compared with the RETOUCH leaderboard for IRF and PED segmentation could be due to the differences in image quality and the heterogeneous conditions within the clinical trial setting (e.g., multiple study sites, imaging technicians, patient factors). In contrast, the images in RETOUCH were selected for exceptionally high quality. The challenges of manually identifying and correctly delineating these features on SD-OCT and distinguishing them from adjoining normal retina, gliosis, or other lesions in clinical trials and real-world settings cannot be underestimated. Therefore, an ML approach using a variety of images of different quality to train the model may be generalizable to a wider variety of data.

## 5. Limitations

ML is always impacted by variability and biases in the training data because the annotation of images is performed manually. Although two graders annotated the OCT dataset, each image was only annotated by one of them, potentially leading to a bias. Additionally, FA assessment (the previous ‘reference standard’ for CNV assessment), is subject to reader interpretation, however, here two graders assessed each FA image, with adjudication as needed. Additionally, the scope of the current work is limited to classification of CNV on FA as predominantly classic, minimally classic, and occult phenotypes. As information about other CNV subtypes, such as retinal angiomatous proliferation and PCV, was not available in HARBOR, these phenotypes could not be evaluated. Furthermore, novel SD-OCT classification and terminology suggested by the Consensus on Neovascular Age-Related Macular Degeneration Nomenclature (CONAN) group [12] were not available for our data; therefore, comparison with an OCT-based classification system was not possible. Moreover, as we restricted our selection of non-CNV eyes to fellow eyes without advanced AMD or other pathologies, this may not be representative of what would be encountered in the real world, and prospective validation in a broader population would be needed. Finally, features such as intraretinal hyperreflective foci have not been included in this model, as their role in diagnosis and prognosis have yet to be established [34].

## 6. Conclusions

Our study shows that using ML on SD-OCT images is sufficiently accurate to detect and classify nAMD. This work highlights the reduced need of FA and provides an automated alternative to manual reading of images at baseline. This in turn limits the variety of imaging data sources from which reads are drawn, reduces the need for multiple human graders, and minimizes the risk of inconsistencies in the diagnoses. Finally, automating the read will also help with a major milestone of algorithmic models, which is to streamline and standardize diagnostic processes.

## Figures and Tables

**Table 1:** Segmentation Performance—DICE Scores for the three fluids (IRF, SRF, PED), and SHRM.

Type	*N* (Total)	*N* (Train/Valid)	Validation DICE Mean (SD)
SRF	700	557/143	0.67 (0.05)
IRF	935	694/241	0.46 (0.12)
PED	622	508/114	0.63 (0.07)
SHRM	760	312/65	0.71 (0.06)

**Table 2:** Human Readouts vs. Automated Readouts for AVENUE. Many automated readouts were generated. This table demonstrates that there was a high correlation between manual readouts that most clearly correspond to the automated [MA1] readouts.

Reading Center Feature	Automated Feature	Spearman r
CENT RET THICK µm	Central subfield thickness IB RPE-to-ILM 0.5 mm min	0.84
CENT RET/LESION THICK µm	Central subfield thickness BM-to-ILM 0.5 mm min	0.79
CENT SUBFIELD THICK ILM-RPE µm	Central subfield thickness IB RPE-to-ILM 0.5 mm mean	0.93
CUBE VOL ILM-RPE mm 3.0 mm	Central subfield volume IB RPE-to-ILM 3.0 mm	0.90
LESION THICK µm	Central subfield thickness BM-to-ILM 3.0 mm max	0.83
PED THICK µm	C-scan height PED 3.0 mm	0.71
SUBRET FLUID THICK µm	C-scan height SRF 3.0 mm	0.61

**Table 3:** Diagnostic Accuracy of the Algorithm in Detecting FA-Defined CNV Phenotype on SD-OCT; Cross-validation results report best-tuned performance across the parameter grid. External validation reports unbiased performance against hold-out data.

Outcome	Balance	FP/FN	Cutoff	Sensitivity	Specificity	AUROC (95% CI)
Cross-Validated Performance for CNV vs. No CNV on HARBOR (No Parameter Tuning)						
Any CNV vs. none	1037/531	23/15	0.3815	0.99	0.98	1.00 (0.99–1.00)
Predominantly classic vs. none	163/531	1/7	0.9996	0.99	1.00	1.00 (1.00–1.00)
Minimally classic vs. none	492/531	10/10	0.6227	0.98	0.98	1.00 (0.99–1.00)
Minimally classic + predominantly classic vs. none	653/531	14/6	0.1831	0.99	0.98	0.99 (0.99–1.00)
Occult vs. none	382/531	17/24	0.9785	0.96	0.96	0.99 (0.99–1.00)
Holdout Performance as Measured on 15% of HARBOR						
Minimally classic + predominantly classic vs. occult	104/56	20/19	0.4036	0.67	0.81	0.81 (0.73–0.88)
Predominantly classic vs. minimally classic	22/82	22/8	0.7051	0.74	0.65	0.73 (0.61–0.85)
Occult vs. minimally classic	55/81	25/16	0.5390	0.72	0.70	0.70 (0.60–0.79)
Best-Tuned Performance on HARBOR for Predominantly Classic vs. Occult						
Predominantly classic vs. occult	163/382	32/41	0.6110	0.89	0.81	0.91 (0.89–0.94)
External Performance on AVENUE for Predominantly Classic vs. Occult						
Predominantly classic vs. occult	126/39	7/24	0.8058	0.81	0.84	0.88 (0.82–0.95)

**Table 4:** Resampling performance.

ROC	Sens	Spec	Resample
0.953	0.750	0.948	Fold 1
0.930	0.641	0.957	Fold 2
0.901	0.840	0.880	Fold 3
0.900	0.568	0.960	Fold 4
0.921	0.786	0.950	Fold 5

**Table 5:** Contingency table, counting all combinations of the predicted versus observed.

Predicted	Observed	*n*
POS (OCCULT)	POS (OCCULT)	341
NEG	NEG	131
POS (OCCULT)	NEG	32
NEG	POS (OCCULT)	41

**Table 6:** The features from Figure 4B in descending order of importance. BM, Bruch’s membrane; HFL, Henle’s fiber layer; IB, inner boundary; ILM, inner limiting membrane; max, maximum; OB, outer boundary; OPL, outer plexiform layer; PED, pigment epithelial detachment; ROC, receiver operating characteristic; RPE, retinal pigment epithelium; SHRM, subretinal hyperreflective material; and SRF, subretinal fluid.

Rank	Feature Name
1	C-scan volume SHRM 1.5 mm
2	C-scan volume SHRM 3 mm
3	C-scan volume SHRM 0.5 mm
4	C-scan volume PED 1.5 mm
5	Central subfield thickness BM-to-OB_RPE 0.5 mm max
6	C-scan width SHRM 3 mm
7	C-scan width SHRM 1.5 mm
8	Central subfield thickness BM-to-OB_RPE 1.5 mm max
9	C-scan height PED 0.5 mm
10	Central subfield thickness IB_RPE-to-OPL-HFL 1.5 mm max
11	C-scan volume PED 3 mm
12	Central subfield volume BM-to-OB_RPE 0.5 mm
13	Central subfield thickness BM-to-OB_RPE 1.5 mm mean
14	Central subfield volume BM-to-OB_RPE 1.5 mm
15	Central subfield thickness BM-to-OB_RPE 0.5 mm mean
16	Central subfield thickness IB_RPE-to-OPL-HFL 3.0 mm max
17	Central subfield thickness BM-to-IB_RPE 1.5 mm max
18	Central subfield volume IB_RPE-to-ILM 0.5 mm
19	C-scan width SRF 0.5 mm
20	Central subfield thickness IB_RPE-to-ILM 0.5 mm mean

**Table 7:** The top 20 features from Figure 6 in descending order of importance.

Rank	Feature Name
1	C-scan volume SHRM 1.5 mm
2	C-scan volume SHRM 3.0 mm
3	C-scan volume SHRM 0.5 mm
4	C-scan volume PED 1.5 mm
5	C-scan volume PED 3.0 mm
6	Central subfield thickness BM-to-OB_RPE 0.5 mm max
7	C-scan width SHRM 3.0 mm
8	C-scan height PED 1.5 mm
9	Central subfield thickness IB_RPE-to-ILM 1.5 mm max
10	Central subfield volume BM-to-OB_RPE 1.5 mm
11	Central subfield thickness IB_RPE-to-ILM 0.5 mm max
12	C-scan height IRF 3.0 mm
13	C-scan height PED 3.0 mm
14	Central subfield thickness BM-to-ILM 1.5 mm max
15	Central subfield thickness BM-to-OB_RPE 1.5 mm max
16	Central subfield thickness BM-to-ILM 3.0 mm min
17	Central subfield thickness OPL-HFL-to-ILM 0.5 mean
18	Central subfield thickness BM-to-ILM 0.5 mm max
19	Central subfield thickness BM-to-OB_RPE 0.5 mm mean
20	Central subfield thickness BM-to-ILM 3.0 mm max

**Table 8:** Contingency table, counting all combinations of the predicted versus observed. Predicted indicates class predicted by the model; observed indicates class as graded on FA; and n indicates the number of samples.

Predicted	Observed	*n*
POS (OCCULT)	POS (OCCULT)	102
NEG	NEG	32
POS (OCCULT)	NEG	7
NEG	POS (OCCULT)	24

## Data Availability

Qualified researchers may request access to individual patient-level data through the clinical study data request platform (https://vivli.org/, accessed on 7 June 2021). Further details on Roche’s criteria for eligible studies are available here (https://vivli.org/members/ourmembers/, accessed on 7 June 2021). For further details on Roche’s Global Policy on the Sharing of Clinical Information and how to request access to related clinical study documents, see here (https://www.roche.com/research_and_development/who_we_are_how_we_work/clinical_trials/our_commitment_to_data_sharing.htm, accessed on 7 June 2021).

## Supplementary Materials

The following are available online at https://www.mdpi.com/article/10.3390/jpm11060524/s1. Figure S1: non-nested cross-validation, Figure S2, Table S1: Definitions used for the annotations, Table S2: Detailed feature list, Supplementary Methods: Details on ML methodology.
