# Supplementary material for: Accuracy of a Machine-Learning Algorithm for Detecting and Classifying Choroidal Neovascularization on Spectral-Domain Optical Coherence Tomography
Source: J Pers Med. 2021 Jun 8;11(6):524. doi: 10.3390/jpm11060524 (PMC8227725; doi:10.3390/jpm11060524)
Supplement: Supplementary file 1 [file jpm-11-00524-s001.zip › jpm-1212001-supplementary.pdf]

## SUPPLEMENTARY METHODS

Machine Learning Methodology

The 105 SD-OCT features from HARBOR were inspected for their utility to predict various binary outcomes (see Results section in the main text for outcome definitions), by using the Random Forest algorithm with the full 105-feature set and 100% of HARBOR data in a five-fold cross validation. No hyper-parameters of the algorithm were tuned. In case a mean performance of 99% AUROC or higher was reached, this performance is reported. This was the case for all settings where we tested CNV vs no CNV.

For minimally classic plus predominantly classic versus occult, predominantly classic versus minimally classic, and occult versus minimally classic, the process was repeated with 85% of the HARBOR data and the least important features were recursively eliminated in steps of five, repeating cross-validation with the reduced set at each such step and re-ranking features. Those features were removed at each step that ranked lowest according to the feature importance measure provided by the Random Forest algorithm. The best feature set thus determined was recorded. Next, the CatBoost algorithm,[1] a gradient-boosting ML algorithm, was used to associate patterns in the best feature set with the outcome. In order to identify good hyper-parameter settings, a 10 × 10 grid over a range of values for tree depth and learning rate was exhaustively searched for optimal value combinations within each learning task, using categorical cross-entropy as the loss function. At each parameter combination (point in the grid), five-fold cross-validation was used to estimate performance for the current combination, and a model trained on the pooled data (across the five folds) using the best hyper-parameter values was used to predict the 15% held out data and report the performance (Fig. S1).

For the case of classifying predominantly classic versus occult CNV, a single model was established on 100% of the HARBOR data as described above and used to predict the AVENUE data and report the performance (Fig. S2).

**SUPPLEMENTARY FIGURE S1.** Validation methodology when splitting HARBOR. CV, cross validation; RFE, Recursive feature elimination.

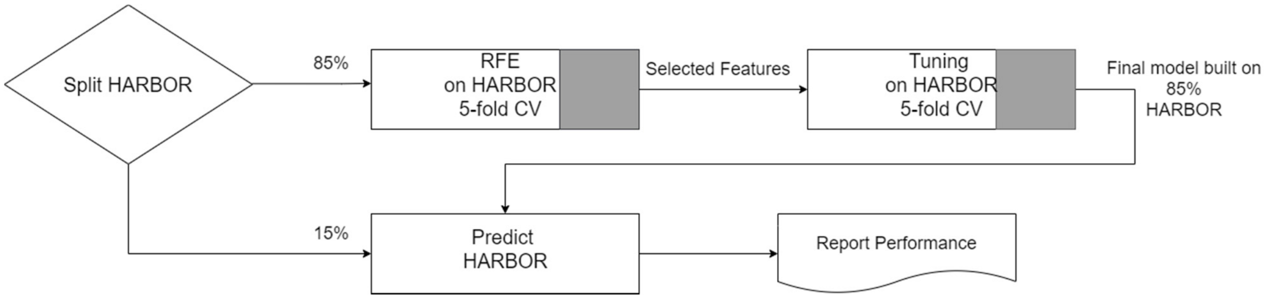

**SUPPLEMENTARY FIGURE S2.** External validation methodology (training on HARBOR, validation on AVENUE). CV, cross validation; RFE, Recursive feature elimination.

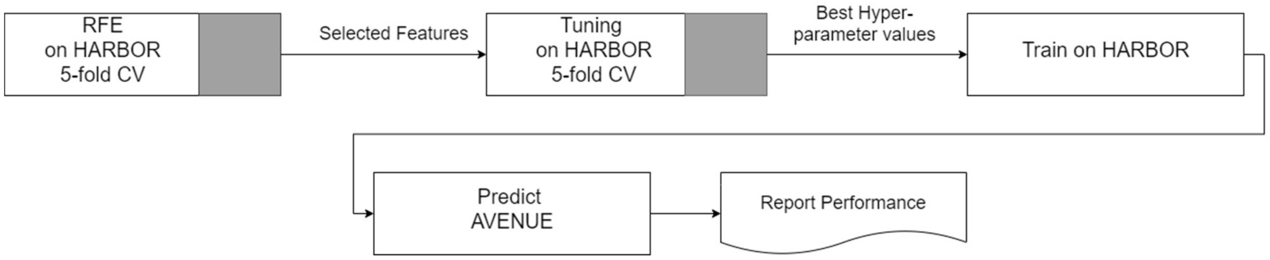

**SUPPLEMENTARY TABLE S1.** Definitions of Annotations.

| Type | Definition |
|---|---|
| SRF | Well-defined hyporeflective spaces between the neural retina and RPE |
| IRF | Circular hypo-reflective spaces within the neural retina that are individually equal to or more than 50 μm in size |
| PED | Elevation of the RPE between the RPE and Bruch's membrane |
| SHRM | A morphological feature seen on OCT as hyperreflective material located external to the retina, and internal to the RPE |

IRF, intraretinal fluid; OCT, optical coherence tomography; PED, pigment epithelial detachment; RPE, retinal pigment epithelium; SHRM, subretinal hyperreflective material; SRF, subretinal fluid.

**SUPPLEMENTARY TABLE S2.** SD-OCT Features Reference: Overview of All 105 Automatically Generated Features

| Feature Type | Features Described (with respect to ETDRS ring radius in mm) |
|---|---|
| Fluidic summary statistics central subfield | C-scan width IRF 0.5 mm |
| Fluidic summary statistics central subfield | C-scan height IRF 0.5 mm |
| Fluidic summary statistics central subfield | C-scan width IRF 1.5 mm |
| Fluidic summary statistics central subfield | C-scan height IRF 1.5 mm |
| Fluidic summary statistics central subfield | C-scan width IRF 3.0 mm |
| Fluidic summary statistics central subfield | C-scan height IRF 3.0 mm |
| Fluidic summary statistics central subfield | C-scan width SRF 0.5 mm |
| Fluidic summary statistics central subfield | C-scan height SRF 0.5 mm |
| Fluidic summary statistics central subfield | C-scan width SRF 1.5 mm |
| Fluidic summary statistics central subfield | C-scan height SRF 1.5 mm |
| Fluidic summary statistics central subfield | C-scan width SRF 3.0 mm |
| Fluidic summary statistics central subfield | C-scan height SRF 3.0 mm |
| Fluidic summary statistics central subfield | C-scan width PED 0.5 mm |
| Fluidic summary statistics central subfield | C-scan height PED 0.5 mm |
| Fluidic summary statistics central subfield | C-scan width PED 1.5 mm |
| Fluidic summary statistics central subfield | C-scan height PED 1.5 mm |
| Fluidic summary statistics central subfield | C-scan width PED 3.0 mm |
| Fluidic summary statistics central subfield | C-scan height PED 3.0 mm |
| Fluidic summary statistics central subfield | C-scan width SHRM 0.5 mm |
| Fluidic summary statistics central subfield | C-scan height SHRM 0.5 mm |
| Fluidic summary statistics central subfield | C-scan width SHRM 1.5 mm |
| Fluidic summary statistics central subfield | C-scan height SHRM 1.5 mm |
| Fluidic summary statistics central subfield | C-scan width SHRM 3.0 mm |

| | |
|---|---|
| Fluidic summary statistics central subfield | C-scan height SHRM 3.0 mm |
| Fluidic volume subfield | C-scan volume IRF 0.5 mm |
| Fluidic volume subfield | C-scan volume SRF 0.5 mm |
| Fluidic volume subfield | C-scan volume PED 0.5 mm |
| Fluidic volume subfield | C-scan volume SHRM 0.5 mm |
| Fluidic volume subfield | C-scan volume IRF 1.5 mm |
| Fluidic volume subfield | C-scan volume SRF 1.5 mm |
| Fluidic volume subfield | C-scan volume PED 1.5 mm |
| Fluidic volume subfield | C-scan volume SHRM 1.5 mm |
| Fluidic volume subfield | C-scan volume IRF 3.0 mm |
| Fluidic volume subfield | C-scan volume SRF 3.0 mm |
| Fluidic volume subfield | C-scan volume PED 3.0 mm |
| Fluidic volume subfield | C-scan volume SHRM 3.0 mm |
| Layer thickness central subfield | Central subfield thickness IB_RPE-to-ILM 0.5 mm mean |
| Layer thickness central subfield | Central subfield thickness IB_RPE-to-ILM 0.5 mm max |
| Layer thickness central subfield | Central subfield thickness IB_RPE-to-ILM 0.5 mm min |
| Layer thickness central subfield | Central subfield thickness IB_RPE-to-ILM 1.5 mm mean |
| Layer thickness central subfield | Central subfield thickness IB_RPE-to-ILM 1.5 mm max |
| Layer thickness central subfield | Central subfield thickness IB_RPE-to-ILM 1.5 mm min |
| Layer thickness central subfield | Central subfield thickness IB_RPE-to-ILM 3.0 mm mean |

| | |
|---|---|
| Layer thickness central subfield | Central subfield thickness IB_RPE-to-ILM 3.0 mm max |
| Layer thickness central subfield | Central subfield thickness IB_RPE-to-ILM 3.0 mm min |
| Layer thickness central subfield | Central subfield thickness BM-to-ILM 0.5 mm mean |
| Layer thickness central subfield | Central subfield thickness BM-to-ILM 0.5 mm max |
| Layer thickness central subfield | Central subfield thickness BM-to-ILM 0.5 mm min |
| Layer thickness central subfield | Central subfield thickness BM-to-ILM 1.5 mm mean |
| Layer thickness central subfield | Central subfield thickness BM-to-ILM 1.5 mm max |
| Layer thickness central subfield | Central subfield thickness BM-to-ILM 1.5 mm min |
| Layer thickness central subfield | Central subfield thickness BM-to-ILM 3.0 mm mean |
| Layer thickness central subfield | Central subfield thickness BM-to-ILM 3.0 mm max |
| Layer thickness central subfield | Central subfield thickness BM-to-ILM 3.0 mm min |
| Layer thickness central subfield | Central subfield thickness BM-to-OB_RPE 0.5 mm mean |
| Layer thickness central subfield | Central subfield thickness BM-to-OB_RPE 0.5 mm max |

| | |
|---|---|
| Layer thickness central subfield | Central subfield thickness BM-to-OB_RPE 0.5 mm min |
| Layer thickness central subfield | Central subfield thickness BM-to-OB_RPE 1.5 mm mean |
| Layer thickness central subfield | Central subfield thickness BM-to-OB_RPE 1.5 mm max |
| Layer thickness central subfield | Central subfield thickness BM-to-OB_RPE 1.5 mm min |
| Layer thickness central subfield | Central subfield thickness BM-to-OB_RPE 3.0 mm mean |
| Layer thickness central subfield | Central subfield thickness BM-to-OB_RPE 3.0 mm max |
| Layer thickness central subfield | Central subfield thickness BM-to-OB_RPE 3.0 mm min |
| Layer thickness central subfield | Central subfield thickness BM-to-IB_RPE 0.5 mm mean |
| Layer thickness central subfield | Central subfield thickness BM-to-IB_RPE 0.5 mm max |
| Layer thickness central subfield | Central subfield thickness BM-to-IB_RPE 0.5 mm min |
| Layer thickness central subfield | Central subfield thickness BM-to-IB_RPE 1.5 mm mean |
| Layer thickness central subfield | Central subfield thickness BM-to-IB_RPE 1.5 mm max |
| Layer thickness central subfield | Central subfield thickness BM-to-IB_RPE 1.5 mm min |

| | |
|---|---|
| Layer thickness central subfield | Central subfield thickness BM-to-IB_RPE 3.0 mm mean |
| Layer thickness central subfield | Central subfield thickness BM-to-IB_RPE 3.0 mm max |
| Layer thickness central subfield | Central subfield thickness BM-to-IB_RPE 3.0 mm min |
| Layer thickness central subfield | Central subfield thickness IB_RPE-to-OPL-HFL 0.5 mm mean |
| Layer thickness central subfield | Central subfield thickness IB_RPE-to-OPL-HFL 0.5 mm max |
| Layer thickness central subfield | Central subfield thickness IB_RPE-to-OPL-HFL 0.5 mm min |
| Layer thickness central subfield | Central subfield thickness IB_RPE-to-OPL-HFL 1.5 mm mean |
| Layer thickness central subfield | Central subfield thickness IB_RPE-to-OPL-HFL 1.5 mm max |
| Layer thickness central subfield | Central subfield thickness IB_RPE-to-OPL-HFL 1.5 mm min |
| Layer thickness central subfield | Central subfield thickness IB_RPE-to-OPL-HFL 3.0 mm mean |
| Layer thickness central subfield | Central subfield thickness IB_RPE-to-OPL-HFL 3.0 mm max |
| Layer thickness central subfield | Central subfield thickness IB_RPE-to-OPL-HFL 3.0 mm min |
| Layer thickness central subfield | Central subfield thickness OPL-HFL-to-ILM 0.5 mm mean |

| | |
|---|---|
| Layer thickness central subfield | Central subfield thickness OPL-HFL-to-ILM 0.5 mm max |
| Layer thickness central subfield | Central subfield thickness OPL-HFL-to-ILM 0.5 mm min |
| Layer thickness central subfield | Central subfield thickness OPL-HFL-to-ILM 1.5 mm mean |
| Layer thickness central subfield | Central subfield thickness OPL-HFL-to-ILM 1.5 mm max |
| Layer thickness central subfield | Central subfield thickness OPL-HFL-to-ILM 1.5 mm min |
| Layer thickness central subfield | Central subfield thickness OPL-HFL-to-ILM 3.0 mm mean |
| Layer thickness central subfield | Central subfield thickness OPL-HFL-to-ILM 3.0 mm max |
| Layer thickness central subfield | Central subfield thickness OPL-HFL-to-ILM 3.0 mm min |
| Layer volume central subfield | Central subfield volume IB_RPE-to-ILM 0.5 mm |
| Layer volume central subfield | Central subfield volume OPL-HFL-to-ILM 0.5 mm |
| Layer volume central subfield | Central subfield volume BM-to-ILM 0.5 mm |
| Layer volume central subfield | Central subfield volume BM-to-OB_RPE 0.5 mm |
| Layer volume central subfield | Central subfield volume BM-to-IB_RPE 0.5 mm |
| Layer volume central subfield | Central subfield volume IB_RPE-to-ILM 1.5 mm |

| | |
|---|---|
| Layer volume central subfield | Central subfield volume OPL-HFL-to-ILM 1.5 mm |
| Layer volume central subfield | Central subfield volume BM-to-ILM 1.5 mm |
| Layer volume central subfield | Central subfield volume BM-to-OB_RPE 1.5 mm |
| Layer volume central subfield | Central subfield volume BM-to-IB_RPE 1.5 mm |
| Layer volume central subfield | Central subfield volume IB_RPE-to-ILM 3.0 mm |
| Layer volume central subfield | Central subfield volume OPL-HFL-to-ILM 3.0 mm |
| Layer volume central subfield | Central subfield volume BM-to-ILM 3.0 mm |
| Layer volume central subfield | Central subfield volume BM-to-OB_RPE 3.0 mm |
| Layer volume central subfield | Central subfield volume BM-to-IB_RPE 3.0 mm |

BM, Bruch's membrane; HFL, Henle's fiber layer; IRF, intraretinal fluid; ILM, inner limiting membrane; max, maximum; min, minimum; RPE, retinal pigment epithelium; IB_RPE, inner boundary of RPE; OB_RPE, outer boundary of RPE; OPL, outer plexiform layer; PED, pigment epithelial detachment; SD-OCT, spectral-domain optical coherence tomography; SHRM, subretinal hyperreflective material; SRF, subretinal fluid.

## Reference


1. Prokhorenkova L, Gusev G, Vorobev A, Dorogush AV, Gulin A. CatBoost: unbiased boosting with categorical features. In: Advances in Neural Information Processing Systems 31 (NIPS 2018). https://papers.nips.cc/paper/7898-catboost-unbiased-boosting-with-categorical-features. Accessed March 24, 2020.
